# Fever, Inflammatory Response, and a Persistent Rash

**DOI:** 10.31138/mjr.33.3.368

**Published:** 2022-09-30

**Authors:** Dimitrios Daoussis, Pantelis Kraniotis, Nikolaos Maltezos

**Affiliations:** 1 Department of Rheumatology, University of Patras Medical School, Patras University Hospital, Patras, Greece,; 2 Department of Radiology, University of Patras Medical School, Patras University Hospital,; 3 Dermatologist, Patras, Greece

**Keywords:** autoinflammatory diseases, anakinra, Schnitzler syndrome, IL-1

**Figure 1.**
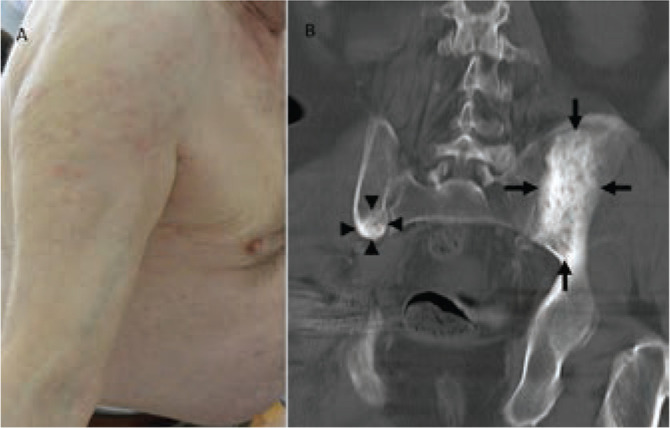
**(a)** Skin rash with urticaria-like features. **(b)** Reformatted coronal oblique CT image (bone windows) at the level of the sacroiliac joints. Centred at the left ilium, there is a predominantly sclerotic lesion with lace-like pattern, narrow zone of transition and relatively well-defined margins (arrows). Note the presence of a similar smaller lesion on the right iliac bone (arrowheads).

A 68-year-old Caucasian male was referred to the Rheumatology Department with the clinical suspicion of adult-onset Still’s disease (AOSD) since the patient had fever, high inflammatory markers, a rash and an extensive work up had excluded infectious or neoplastic causes. Twelve months ago, the patient first developed a non-pruritic rash on his torso and arms that recurred frequently. During the last few months, the patient developed fever and significant weight loss, therefore a series of investigations was performed. Lab tests revealed an ESR of 130mm/h, a 15-fold elevation of CRP, leucocytosis with normal complement levels, and no autoantibodies. A full body CT and temporal artery biopsy were unremarkable. An IgMκ monoclonal gammopathy was found and the patient was admitted to hospital for further investigations with the initial suspicion being a hematologic malignancy. An extensive evaluation including bone marrow biopsy and PET/CT ruled out lymphomas, myeloproliferative disorders or plasma cell dyscrasias whereas a thorough work up for infectious diseases was negative. When we first evaluated the patient, we noticed that the rash had urticaria-like features and was not compatible with AOSD (**Figure 1A**). Ferritin levels were within normal limits despite the robust inflammatory response making the diagnosis of AOSD even more unlikely. Since the rash was the first sign of the disease, a full review of the patient’s record was performed in collaboration with dermatology colleagues. The combination of an urticarial rash, IgMκ monoclonal gammopathy, fever and inflammatory response pointed towards Schnitzler’s syndrome, a rare but potentially underdiagnosed, autoinflammatory disease of unknown aetiology. Notably, extensive osteosclerotic lesions in the pelvis were noted on imaging, also suggestive of the disease (**Figure 1B**). The patient started treatment with anakinra and exhibited a dramatic response with disappearance of skin lesions, and normalization of inflammatory markers.

